# The treatment of established non-union of the proximal humerus using the Polarus locking intramedullary nail

**DOI:** 10.4103/0973-6042.59970

**Published:** 2009

**Authors:** Steven W. Hamilton, Kevin S. Baird

**Affiliations:** Department of Orthopaedics, Aberdeen Royal Infirmary, Aberdeen, Scotland, U.K; 1 Raigmore Hospital, Inverness, Scotland, U.K

**Keywords:** Non-union, proximal humerus, locking nail

## Abstract

**Introduction:**

Non-union following fracture of the proximal humerus is not uncommon, particularly in the elderly. This can be associated with significant morbidity due to pain, instability and functional impairment. The Polarus device (Acumed) is a locked, antegrade intramedullary nail designed to stabilize displaced 2-, 3- and 4-part fractures of the proximal humerus. We report our experience with the Polarus nail for the treatment of established non-union of the proximal humerus.

**Materials and Methods:**

A total of 7 Polarus nails were inserted for the treatment of non-union of the proximal humerus between June 2000 and July 2007. Each fracture site was opened, debrided, stabilized with a Polarus nail and then grafted with autologous cancellous iliac crest bone. The time between injury and surgery ranged from 6 to 102 months. One patient had undergone previous fixation of her fracture using Rush intramedullary rods. All patients were females, and mean age at surgery was 63.6 years (range, 49-78 years). A retrospective review of notes and radiographs was carried out. Patients were reviewed at varying intervals postoperatively (range, 13-68 months) and assessed using the Constant shoulder-scoring system.

**Results:**

All un-united fractures progressed to union. There were no wound complications and no postoperative nerve palsies. Functional outcome was good, even in those cases with a long interval between injury and surgery. The mean Constant score was 63 (range, 54-81). Migration of a single proximal locking screw was seen in 2 patients, and these screws required removal at 5 and 12 months, respectively, postoperatively.

**Conclusion:**

In our experience, a locked proximal humeral nail used in conjunction with autologous bone grafting is an excellent device for the treatment of proximal humerus non-unions.

## INTRODUCTION

Fractures of the proximal humerus account for approximately 5% of all fractures.[1,2] Their incidence increases rapidly beyond the age of 50 years, and they are twice as common in women than in men.[1,3,4] Most heal uneventfully; however, non-union is not uncommon, particularly in the elderly. The incidence of non-union in this group of patients can be as high as 23%.[5] Risk factors for non-union include multiple medical comorbidities, smoking, fracture comminution, alcohol abuse and loss of fixation in osteoporotic bone.[6,7] Non-union of the proximal humerus results in significant morbidity due to pain, instability and functional impairment.[7,8] Several operative techniques have been used to treat proximal humerus non-unions with varying success.[6,8‐17] Treatment of non-union of the proximal humerus continues to be an orthopedic challenge.

The Polarus device (Acumed) is a locked, antegrade intramedullary nail designed to stabilize displaced 2-, 3- and 4-part fractures of the proximal humerus. [18‐23] We report our own experience with the Polarus nail in the treatment of established non-union of the proximal humerus.

## MATERIALS AND METHODS

Seven patients underwent insertion of a Polarus nail for non-union of the proximal humerus between June 2000 and July 2007 at Raigmore Hospital, Inverness, Scotland, U.K. The second author exclusively treated all 7 patients. Each non-union was exposed via a deltopectoral approach. Fibrous tissue was excised and the bone ends were debrided. In each patient, the proximal fragment was found to retain good soft tissue attachments, with bleeding observed from bone ends. The humeral head was therefore considered viable. A Polarus nail was inserted, locked proximally with up to 4 screws and then distally with 2 screws. The bony defect was then packed with autologous cancellous bone graft harvested from the ipsilateral iliac crest. A structural cortico-cancellous graft or bone peg was not used in any of the cases. One patient had undergone 2 previous operations for persistent non-union. All patients were female with a mean age of 63.6 years (range, 49-78 years). Established non-union was defined as no clinical or radiological evidence of bony union at 6 months or more after fracture. The time between injury and surgery ranged from 6 to 102 months. In each case, the initial injury was a displaced, 2-part surgical neck of humerus fracture. A retrospective review of notes and radiographs was carried out. Functional assessment of each patient was performed using the Constant shoulder score. Follow-up interval ranged from 13 to 68 months. The clinical data is summarized in Table 1.

**Table 1:** Clinical data for 7 patients with established non-union of the proximal humerus treated by Polarus intramedullary nail with autologous iliac crest bone grafting

Gender	Age	Previous operation	Time between injury and nail (m)	Follow-up time (m)	Constant shoulder score	Complication
F	49	N	6	16	81	Single screw migration
F	64	N	7	16	59	N
F	67	N	11	13	70	N
F	57	N	11.5	46	58	N
F	78	N	12	65	65	N
F	64	N	16.5	13	54	N
F	66	Rush rods	102	68	54	Single screw migration

## RESULTS

**Figure 1:**
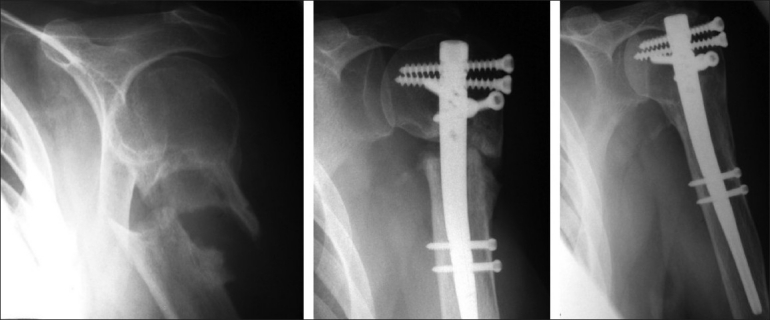
Progression of a proximal non-union to union after Polarus nailing and autologous cancellous bone grafting

All un-united fractures progressed to union, and a typical example is shown in Figure 1. Functional outcome was good even in those patients with a long interval between injury and surgery. The average Constant shoulder score was 63 (range, 54-81). There were no wound complications, no postoperative neurovascular problems and no significant donor site morbidities. Migration of a single proximal screw was observed in 2 patients, which resulted in a localized prominence with mild discomfort in the lateral deltoid area. These symptoms resolved following removal of the screws, under local anesthesia, at 5 and 12 months, postoperatively. One patient had bilateral proximal humerus non-unions that had resulted from non-simultaneous injuries. One was treated by cemented hemiarthroplasty and the other by a Polarus nail with autologous bone grafting. The side with the hemiarthroplasty did not function as well when compared to the nailed side.

## DISCUSSION

Non-union of the proximal humerus is a debilitating problem which can be difficult to treat. Various operative treatments with varying rates of success have been described.

Complications following Rush rods with autologous bone grafting include persistent non-union and symptomatic metalwork migration. Scheck[6] reported good results in 5 patients treated with this method. However, Nayak *et al*. [14] reported poor results in a similar group of 10 patients. Non-union persisted in 2 patients, osteonecrosis of the humeral head developed in 2 patients, while 8 patients required removal of the Rush rods for mechanical impingement.

Hemiarthroplasty provides good pain relief, but function often remains limited despite rotator cuff reconstruction.[8,11,14]

Plate fixation with autologous bone grafting has been shown to give better results when compared with Rush rod fixation or hemiarthroplasty.[9,11,12,15] However, soft tissue stripping during plate fixation may increase the risk of osteonecrosis of the humeral head.[7] Sturzenegger *et al*.[24] reported osteonecrosis in 34% of patients treated with *t*-plate fixation, although this may have been due to the severity of the fracture. Symptomatic metalwork impingement and soft tissue adhesion to the plate are also recognized complications of this method. However, Walch *et al*.[17] reported a low complication rate and 96% union with plate fixation when combined with intramedullary bone peg insertion and cancellous bone grafting.

The treatment of surgical neck non-unions with a locked intramedullary nail has previously been described.[13] The locking nail used was designed and manufactured at the center where the study took place. Fourteen out of 15 patients united, but there was a high complication rate: 2 patients had iatrogenic fractures, 2 had locking screws misplaced, 2 screws became loose and 1 suffered a postoperative brachial plexus injury.

All 7 un-united fractures in our study progressed to union. As follow-up was at varying intervals, no conclusions have been drawn regarding time to union. Apart from migration of a single proximal locking screw in 2 patients, there were no complications. In our experience, a locked proximal humeral nail in combination with autologous cancellous bone grafting is a safe and reliable technique for the treatment of non-union of the proximal humerus. We believe that the success of this technique is due to several reasons: 1) There is minimal soft tissue stripping of the proximal fragment during insertion, thereby avoiding further vascular compromise of the humeral head; 2) the fracture fixation is load-sharing, the biomechanics of which help stimulate bone formation;[16] 3) proximal locking by up to 4 divergent cancellous screws provides multiplanar stability, allowing early mobilization of the shoulder. When compared with other nails, the Polarus device has been shown to give a more stable and rigid construct between bone and nail.[25] Proximal humerus non-unions are more often a consequence of instability rather than a problem with vascularity. Therefore, in some cases stabilization with a locked proximal humeral nail without bone grafting could be a further treatment option.
